# Enhanced deviant responses in patterned relative to random sound sequences

**DOI:** 10.1016/j.cortex.2018.08.032

**Published:** 2018-12

**Authors:** Rosy Southwell, Maria Chait

**Affiliations:** Ear Institute, University College London, London, UK

**Keywords:** Surprise, Prediction error, Mismatch negativity, Predictive coding, Orbitofrontal cortex

## Abstract

The brain draws on knowledge of statistical structure in the environment to facilitate detection of new events. Understanding the nature of this representation is a key challenge in sensory neuroscience. Specifically, it is unknown whether real-time perception of rapidly-unfolding sensory signals is driven by a coarse or detailed representation of the proximal stimulus history. We recorded electroencephalography brain responses to frequency outliers in regularly-patterned (REG) versus random (RAND) tone-pip sequences which were generated anew on each trial. REG and RAND sequences were matched in frequency content and span, only differing in the specific order of the tone-pips. Stimuli were very rapid, limiting conscious reasoning in favour of automatic processing of regularity. Listeners were naïve and performed an incidental visual task. Outliers within REG evoked a larger response than matched outliers in RAND. These effects arose rapidly (within 80 msec) and were underpinned by distinct sources from those classically associated with frequency-based deviance detection. These findings are consistent with the notion that the brain continually maintains a detailed representation of ongoing sensory input and that this representation shapes the processing of incoming information. Predominantly auditory-cortical sources code for frequency deviance whilst frontal sources are associated with tracking more complex sequence structure.

## 1 Introduction

Detection of new events within a constantly fluctuating sensory input is a fundamental challenge to organisms in dynamic environments. Hypothesized to underlie this process is a continually-refined internal model of the real-world causes of sensations, made possible by exploiting statistical structure in the sensory input (Dayan et al., 1995, Friston and Kiebel, 2009, Rubin et al., 2016, Winkler et al., 2009). Evidence from multiple domains, including speech (Saffran, Aslin, & Newport, 1996), abstract sound sequences (McDermott et al., 2013, Paavilainen, 2013, Saffran et al., 1999), vision (Turk-Browne, Scholl, Chun, & Johnson, 2009) and motor control (Bestmann et al., 2008) reveals sensitivity to environmental statistics, which in turn influences top-down, expectation-driven perceptual processing. When the organism encounters sensory input that is inconsistent with the established internal model, a ‘surprise’ response is generated (Friston, 2005), promoting a rapid reaction to the associated environmental change. Understanding what aspects of stimuli are ‘surprising’, and how they are processed, is therefore central to understanding this network.

The auditory system has been a fertile ground for probing sensory error responses, at multiple levels of the processing hierarchy (Aghamolaei et al., 2016, Ayala et al., 2016, Nelken, 2014, Parras et al., 2017). A common approach involves using a stream of standard sounds to establish a regularity that is occasionally interrupted by ‘deviant’ sounds (Garrido et al., 2009, Garrido et al., 2008, Heilbron and Chait, 2017, Khouri and Nelken, 2015, Näätänen and Alho, 1995). Deviants usually evoke an increased response relative to that measured for the standards (Garrido et al., 2009, Herrmann et al., 2015, Ulanovsky et al., 2003). Since many of the investigated sequences have been very simple, often a repeated tone; neural adaptation is likely a major contributor to the observed deviant responses (Briley and Krumbholz, 2013, Grill-Spector et al., 2006, Nelken, 2014). However, accumulating evidence suggests that at least part of the deviant response arises from neural processes associated with computing ‘surprise’ or detecting a mismatch between expected and actual sensory input (Daikhin and Ahissar, 2012, Khouri and Nelken, 2015, Parras et al., 2017, Taaseh et al., 2011). The underlying network, consistently implicated in these processes, is comprised of bilateral auditory cortex (Heschl's Gyrus and superior temporal gyrus) and right inferior frontal gyrus (Barascud et al., 2016, Chennu et al., 2016, Garrido et al., 2009, Garrido et al., 2008, Heilbron and Chait, 2017, Opitz et al., 2002).

What information is used in calculating surprise? Mounting evidence suggests that the deviant response is shaped by the statistics of the sequence as it unfolds. Garrido, Sahani, and Dolan (2013) demonstrated that MEG responses to probe tones are sensitive to the statistical context (mean and variance of frequency) of randomly generated tone-pip sequences such that larger responses occurred to the same probe tone when presented in a context with low-variance than with high-variance. Rubin et al. (2016) modelled brain responses to two-tone sequences with different probabilities. They demonstrated, in line with conclusions from Garrido et al. (2013), that trial-wise neural responses in auditory cortex are well explained by the probability of occurrence of each tone frequency, calculated from the recent history of the sequence. The models that best fit neural responses were based on a relatively long stimulus history (∼10 tones); but maintained a coarse representation, reflecting a small set of summary statistics.

Most previous work investigating the effect of context on deviant processing has focused on simple, random frequency patterns (Garrido et al., 2013, Herrmann et al., 2015, Khouri and Nelken, 2015). For these signals, a coarse representation, possibly underpinned by adaptation processes (Herrmann et al., 2015, Khouri and Nelken, 2015, May and Tiitinen, 2010), may indeed be sufficient to capture relevant attributes. However, it remains unclear whether the brain also keeps track of a detailed history of past sensory experience. To reveal these processes, the stimulus must contain some structural regularity. Whilst previous research (Koelsch et al., 2000, Koelsch et al., 2016, Maess et al., 2001, Pearce et al., 2010, Vaz Pato et al., 2002), investigated complex sequence structure, the experiments mostly involved fixed patterns and exposure over very long durations, likely reflecting long-term structure learning. In contrast, here we focus on structure which emerges anew in each sequence. We seek to understand whether the brain represents this structure, and identify the underlying brain networks.

**Fig. 1:**
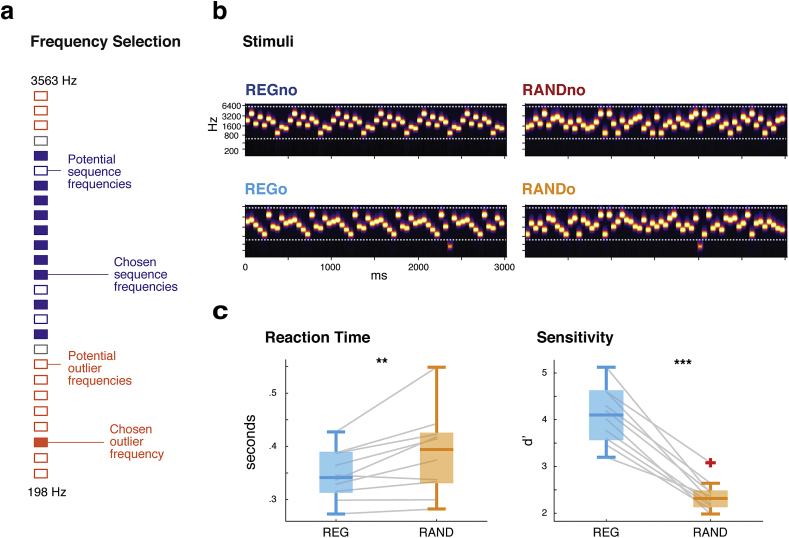
**Stimuli and Behavioural responses. a:** Procedure for selecting frequencies used for each stimulus. From the pool of 26; 13 adjacent values were chosen at random as candidate sequence frequencies (purple); 10 were selected for the sequence. Of the remaining tones; all except the frequencies closest to the sequence could potentially be outliers (orange); and from these a single value was chosen at random to be the outlier on that trial. **b:** Example set of stimuli for the four conditions; these were generated together from the same frequencies in order to match acoustic properties. **c:** Results from the behavioural experiment. **Left:** reaction times to outlier tones. **Right:** sensitivity (d’) to outlier tones. **p < .01, ***p < .001.

We used fast tone-pip sequences, unique on each trial, that occasionally contained a frequency outlier presented outside of the spectral region occupied by the standards. To determine whether the deviant response merely reflects an unexpected change in frequency between the standards and outlier, or whether it is also affected by the specific order of elements in the sequence, we used as standards either regular (REG) or random (RAND) sequences of otherwise matched frequencies (see Fig. 1), such that the frequency span is identical but the precision of the available information regarding successive frequencies is either low (RAND) or high (REG). Notably, the sound sequences were very rapid (20 tones per second) such that conscious reasoning about the sequence order is unlikely to be possible.

Based on the hypothesis that the human brain tracks and evaluates incoming sensory information against the specific pattern established by the sequence context, we expect outlier tones to be more readily detectable in REG than in RAND sequences. The experiments reported below investigate this assertion by measuring deviance-evoked EEG responses in naïve, distracted listeners (Experiment 1) and when listeners actively monitored the sequences for outlier tones (Experiment 2).

## 2 Methods

### 2.1 Stimuli

Stimuli consisted of 50-msec tone pips of varying frequency, arranged in regular (REG) or random (RAND) frequency patterns over a total duration of 3000 msec (60 tones). Frequencies were drawn from a pool of 26 logarithmically-spaced values between 198 and 3563 Hz (12% increase in frequency at each step). To generate each sequence, 13 adjacent frequencies were chosen at random from the larger pool (see Fig. 1a) and then a random subset of 10 of these frequencies were retained, so that all sequences had a similar bandwidth and contained exactly 10 unique frequencies (‘alphabet size’ = 10). REG sequences were generated by permuting the 10 chosen frequencies and then repeating that order six times (Fig. 1b; upper left). Matched RAND sequences were generated by shuffling each REG sequence, with the constraint that no two adjacent tones were the same frequency (Fig. 1b; upper right). Overall, the stimulus generation procedure ensures that REG and RAND sequences are matched in terms of the first-order distribution of tones; the only difference being whether they are arranged in a predictable (REG) or unpredictable (RAND) order.

Half of the sequences (henceforth denoted as REG_O_ and RAND_O_) contained a single frequency ‘outlier’ tone between 1500 and 2750 msec post-onset (latency chosen at random for each stimulus), which is equivalent to a minimum of 3 REG cycles (Fig. 1b; lower panels). Our previous work (Barascud et al., 2016, Southwell et al., 2017) determined that the detection of regularity and the associated brain responses take place between 1 and 2 cycles. A latency of 3 cycles therefore assures that the processing of the regular pattern has stabilized (see also Fig. 3a). The outlier tones replaced the corresponding standard tone. The outlier frequency was either higher or lower than the range spanned by the 10 standard frequencies in the sequence, with a minimum distance of two frequency steps. Throughout the entire set of trials, all 26 frequencies could be outliers or standards. Furthermore, to ensure all ten standard frequencies were approximately equally probable before the outlier, RAND_O_ were generated by shuffling separately before and after the chosen outlier position. Stimuli were generated in matched sets of four (Two containing an outlier: REG_O_, RAND_O_; and two matched ‘controls’ with no outlier: REGno, RANDno), using the same ‘alphabet’ for standards (and the same frequency for the outliers, if applicable). Sequences were unique on each trial and generated anew for each subject.

**Fig. 2:**
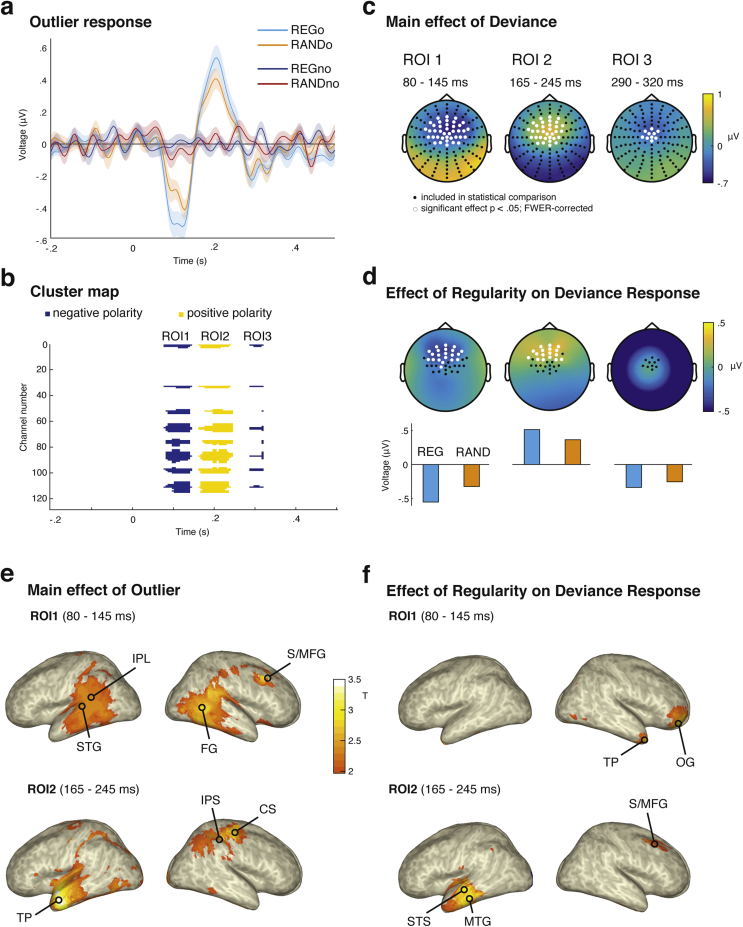
**Deviance-evoked responses. a:** Time-domain response averaged over the 39 central channels which showed a significant deviance response. Shading shows the standard error of the mean over subjects. The three deflections in the response correspond to the three clusters shown in (b). **b:** three time-channel clusters showing a main effect of deviance; i.e., (REGo – REGno) + (RANDo – RANDno) **c:** Topography of the three main-effect ROIs; averaged over the temporal extent of each ROI. **d:** Topography of the effect of regularity: expressed by the contrast (REGo – REGno) – (RANDo – RANDno). Channels included in the statistical analysis are shown in black [these are the significant channels in (c)]. Channels showing an effect of deviance at any point during the cluster are highlighted in white. The average magnitude of the response to REGo and RANDo, within each ROI, is shown in the bar plots below. **e,f:** Source-level activity shown on a template cortical sheet. T-statistic maps thresholded at T = 2. All show average source activity taken over a time-window defined by ROI1 (80–145 msec) and ROI2 (165–245 msec) **e:** Main effect of deviance in ROI1 (**top**) and ROI2 (**bottom**). **f:** Effect of regularity on the deviance response in ROI1 (**top**) and ROI2 msec (**bottom**). Peak T-statistic are indicated. Abbreviations: STG - superior temporal gyrus, IPL - inferior parietal lobule, FG - fusiform gyrus, S/MFG - superior/middle frontal gyrus, TP - temporal pole, IPS - intraparietal sulcus, CS - central sulcus, OG - orbital gyrus, STS - superior temporal sulcus, MTG - middle temporal gyrus.

**Fig. 3:**
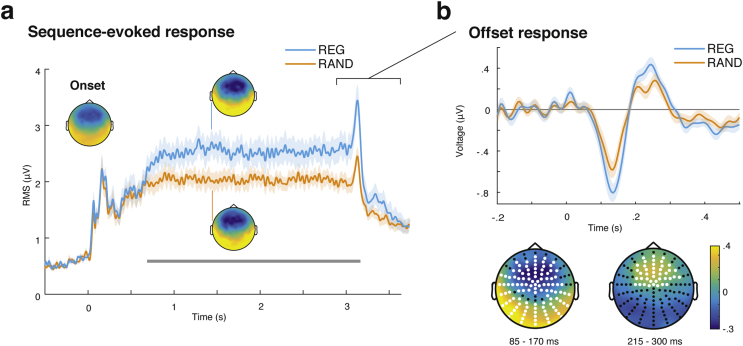
**Sequence-evoked responses. a.** Sequence-evoked response. Shown in the main plot is the root-mean-square (RMS) of the signal over all channels, representing global field power; shading shows the standard error of the mean over subjects. Time period showing significant difference between REG and RAND conditions is indicated by a grey bar. Polarity-resolved topographies (across all channels) are shown for the onset response from 50 to 80 msec (inset; left) and the sustained response (700–3000 msec) to REG (inset; top) and RAND (inset; bottom). **b:** Offset response. **Top:** Evoked response averaged over 58 central channels showing an effect of regularity. **Bottom:** Topography of the response during the two time-windows covering significant clusters for the contrast (REG – RAND); channels showing an effect of deviance at any point during the cluster are highlighted in white.

### 2.2 Experiment 1 – passive EEG responses to frequency-outliers within REG and RAND contexts

#### 2.2.1 Stimuli and procedure

The stimulus set comprised four sequence types: REGo, RANDo, REGno, and RANDno; as described above. These were presented to naïve, distracted listeners whilst their brain activity was recorded with EEG. Each trial was unique and sequences were generated anew for each subject. A total of 600 sequences were presented; 150 of each condition. The session was split into 6 blocks to provide breaks, each with 25 trials per condition presented in a random order. The inter-trial interval (ISI) was jittered between 1100 and 1500 msec. Stimuli were presented with the Psychophysics Toolbox extension in Matlab (Kleiner, Brainard, Pelli, & Ingling, 2007), using EarTone in-ear earphones with the volume set at a comfortable listening level. In order to capture automatic, stimulus-driven deviance detection processes, subjects watched a subtitled film of their choice during the experiment, with the audio muted. They were informed that there would be some sounds played during the session, and were presented with a single example of RANDno as a demonstration; but were instructed to ignore all sounds.

Following the session, subjects were asked the following questions about the sounds they heard:

1. During the EEG experiment, you heard some sounds. How distracting did you find them (1 = not at all, 5 = very distracting all the time)
2. Please describe the sounds briefly – what did you notice?
3. Did you hear any patterns in the sounds?
4. Did you hear any beeps that broke the pattern?

#### 2.2.2 EEG recording and analysis

EEG was recorded using a 128-electrode Biosemi system (Biosemi Active Two AD-box ADC-17, Biosemi, Netherlands) at a sampling rate of 2048 Hz. Data were pre-processed and analysed using Fieldtrip (Oostenveld, Fries, Maris, & Schoffelen, 2010) toolbox for Matlab (2015a, MathWorks). Separate analysis pipelines were used to analyse the whole sequence response (time-locked to sequence onset) and the deviant response (time-locked to the onset of the deviant tone). All filtering was performed with a zero phase-shift Butterworth filter.

**Artefact rejection**: After epoching (see below), epochs containing artefacts were removed on the basis of summary statistics (variance, range, maximum absolute value, z-score, maximum z-score, kurtosis) using Fieldtrip's visual artefact rejection tool. On average 5% of epochs were removed for each subject (range 0–10%). Artefacts related to eye movements, blinks and heartbeat were identified using independent component analysis (ICA). Any channels previously identified as noisy were not included in the ICA procedure.

To analyse the **sequence-evoked response**, data were high-pass filtered at 0.1 Hz (third-order) and divided into 5000-msec epochs (with 1000 msec pre-stimulus-onset and 1000 msec post-offset). After artefact rejection, all data were resampled at 200 Hz with an anti-aliasing lowpass FIR filter, and baseline-corrected relative to the pre-onset interval. Missing bad channels were reconstructed as the average of their immediate neighbours. Subsequently the data were re-referenced to the mean of all channels, averaged over epochs of the same condition, baseline-corrected (200 msec preceding stimulus onset) and low-pass filtered at 30 Hz (fifth-order) for plotting and analysis.

For quantifying the **deviant response**, data were high-pass filtered at 2 Hz (third-order) and divided into 700-msec epochs, with 200 msec baseline and 500 msec following the onset of the outlier tone. The cutoff frequency of 2 Hz was chosen to ensure that any differences in sustained activity between REG and RAND have been eliminated. Conditions without a violation (REGno and RANDno) were epoched relative to the average outlier timing; rounded down to the nearest tone onset, i.e., 2100 msec. These were used as a baseline against which the responses the outlier tones were evaluated. Note that after high pass filtering there was no difference between the REGno and RANDno sequences (see Fig. 2a for illustration and below for statistical analysis). Subsequent analysis steps were identical to those described for the whole sequence analysis (above).

For the **offset response analysis**, the sequence-evoked data were high-pass filtered at 2 Hz, re-aligned into epochs (2800–3500 msec) and baseline-corrected based on the interval 2800–3000 msec. Subsequent analysis steps were identical to those described for the whole sequence analysis (above).

#### 2.2.3 Statistical analysis

To assess the response to the outlier tones **(‘main effect of deviance’)**, we collapsed across context and computed the difference between trials which contained and did not contain an outlier. Formally this is expressed as the contrast: (REGo – REGno) + (RANDo -RANDno). Fieldtrip's cluster-based permutation test, which takes spatial and temporal adjacency into account, was used to correct for multiple comparisons (Maris and Oostenveld, 2007, Oostenveld et al., 2010). The significance threshold was chosen to control family-wise error-rate (FWER) at 5%. This defined three regions of interest (ROI) in time-channel space showing a deviant response. To determine how the **deviant response is affected by regularity (‘effect of regularity’)**, we calculated an orthogonal contrast of the deviance response magnitude by sequence type, formally expressed as (REGo – REGno) – (RANDo – RANDno), for each of the ROIs defined above. Statistical analysis was performed across channels using the same cluster-based permutation test described previously. The same statistical procedure was performed to verify that there was no residual difference in the responses to REGno and RANDno, ensuring that any effect on the deviance response reflects processing of the outlier tone rather than differential processing of the control condition.

The **offset peak** was compared between REG and RAND (collapsed across outlier and no-outlier trials), across the whole scalp and offset epoch, using the same clustering approach as described above for the deviance response.

To characterize the **overall sequence-evoked response** to REG and RAND, the root mean square (RMS) of the evoked potential over all channels was calculated for each time point to give a time-series which reflects the instantaneous power of the evoked response. In the current data, as well as previous studies with similar stimuli, the sustained response is characterised by a large DC-like shift without zero-crossings, and with similar response dynamics in all channels; thus the RMS is a faithful representation of the dynamics in individual channels (see Fig. 3 from Barascud et al., 2016, Southwell et al., 2017). The distribution of RMS across subjects (mean, standard error, confidence interval) was then estimated for each condition using bootstrap resampling across subjects (Efron & Tibshirani, 1993) with 1000 iterations, for plotting of the group average response in Fig. 3a. The significance of the difference in RMS between REG and RAND was assessed using the same cluster-based permutation statistics as for the deviant response, at each time sample, from sequence onset to 500 msec following offset. T-tests (2-tail) were performed using t-statistics computed on clusters in time, and controlled for a family-wise error rate of .05 (Maris & Oostenveld, 2007).

#### 2.2.4 Source analysis

In the absence of individual structural scans, a head model derived from a template MNI brain was used (colin27; as included in the Fieldtrip toolbox) for which the volume conductance model was computed from MRI images using the Boundary Element Method (Fuchs, Kastner, Wagner, Hawes, & Ebersole, 2002). A triangulated cortical sheet, with 5124 vertices, derived from this scan was used as the source model. Source inversion was performed on individual subjects and separately for each condition, using Minimum Norm Estimation (MNE; Dale et al., 2000). Source activity was reconstructed over a time-window spanning 0–300 msec relative to the onset of the outlier. Source data were then averaged within the time intervals 80–145 and 165–245 which correspond to the two ROI time windows in which significant effects were found in time/sensor space. Subsequently, T-statistic maps were computed, within each time window, for the **main effect of deviance**: (REGo + RANDo) > (REGno + RANDno), and the orthogonal **effect of regularity**: (REGo – REGno) > (RANDo – RANDno). Data were interpolated onto an inflated cortical surface for visualisation (Fig. 2e and f) and are presented using a threshold of T = 2. Because the contrasts are motivated by significant effects in the time domain, further statistical inference was not performed to avoid circularity (per Gross et al., 2013). Due to the limited precision afforded by the template-based source modelling used here, we discuss activation patterns in terms of general areas as opposed to specific MNI coordinates.

#### 2.2.5 Participants

Data from 20 paid subjects are reported (age 19–32, mean 22.8 years. 9 female). None participated in the behavioural study (Experiment 2). All (here and in Experiment 2 below) were right handed and reported normal hearing and no history of neurological disorders. One additional subject was excluded from analysis due to excessively noisy data. The experimental protocol for both experiments reported here (Experiment 1 and 2) was approved by the University College London research ethics committee.

### 2.3 Experiment 2 – behavioural sensitivity to frequency-outliers in REG and RAND sequences

#### 2.3.1 Stimuli and procedure

Subjects heard 96 trials each of REGno, RANDno, REGo and RANDo (in random order), and were instructed to respond by button press when they heard a deviant tone. Fourty-eight additional control trials were also included, with the same number and timing of tone pips, but consisting of a single, repeating standard frequency (CTRL). Twenty-four of these contained an outlier tone at least 2 whole tones away from the standard (CTRLo); deviant and standard frequencies were chosen at random for each stimulus. Subjects were instructed to respond by button press as quickly as possible when a deviant tone was detected. Trials were presented in a random order, but the proportion of each condition across each block of 72 trials was kept the same. The testing session was preceded by a practise session of 28 trials; conditions were the same as the main experiment and in the same proportions.

#### 2.3.2 Analysis

Dependent measures are d’ scores (Tanner & Swets, 1954) and response times (RT; measured between the onset time of the outlier and the subject's key press). Trials deviating from the condition-wise mean reaction time by more than 2 SD were excluded; this resulted in exclusion of no more than 6% of trials for each condition. Sensitivity scores (d’) to deviants in each condition were calculated using the hit and false alarm rates. In cases where either rate was 0 or 1, a half trial was (respectively) added or subtracted to the numerator and denominator of the rate calculation; to avoid infinite d’ values.

#### 2.3.3 Participants

10 paid participants took part (age 18–34, mean 24.4 years; 5 female). None participated in the EEG study (Experiment 1).

## 3 Results

### 3.1 Experiment 1 – EEG responses to frequency-outliers within REG and RAND contexts in naïve, passively listening participants

EEG responses were recorded to REG and RAND (Fig. 1) sequences which occasionally contained a frequency outlier. Overall frequency occurrence statistics, taken over the sequence duration or over the entire experimental session, are identical between REG and RAND. The resulting effect is that the context offered by each sequence differs in predictability but not in frequency span. In order to capture automatic, stimulus-driven deviance detection processes, participants were kept naïve and distracted, watching a silent, subtitled movie of their choice.

#### 3.1.1 Post-session reports

Following the EEG experiment, participants were questioned about the sounds presented. Nine out of twenty described hearing some kind of pattern in the sound, for instance ‘repetition’ and ‘alternating high and low sounds’, although these descriptions were usually quite vague, and when pressed to elaborate, none had noticed the distinction between REG and RAND trials. Thirteen subjects reported hearing occasional sounds which broke the pattern, or were otherwise distinctive; and when asked to elaborate, several specified that the pitch of the tones stood out as higher or lower than the rest. This shows that the outliers entered subjects' awareness at least in some cases, although accurate description of the patterning of the sequences was much rarer. The mean rating given for how distracting the sound sequences were overall was 2.2 out of 5, range 1–4; indicating that subjects were moderately distracted by the sound sequences on average, but with considerable variability.

#### 3.1.2 Sequence-evoked EEG responses

Sequence-evoked responses (Fig. 3a) were analysed by pooling across conditions which contained or did not contain an outlier. The standard sequence of auditory onset responses is seen, followed by a rise to a sustained response that persists until stimulus offset. The topography of this response for both REG and RAND is similar to the N1 onset response, namely a fronto-central negativity (see inset topographies; Fig. 2c). The response to REG was significantly greater than that to RAND, from 705 msec after onset until 440 msec after offset (*p* < .001, FWER-corrected). The response to REG diverged from RAND after just 4 tone-pips (200 msec) of the first repeated cycle, demonstrating that the brains of naïve distracted listeners are sensitive to sequence structure, discovering the regularity very rapidly (in fact, as early as expected from an ideal observer see Barascud et al., 2016). Overall this pattern of results entirely replicates previous work (Barascud et al., 2016, Southwell et al., 2017). However, the present stimuli are better controlled for effects of frequency-specific adaptation, by ensuring that REG and RAND have exactly the same frequency content; and by disallowing repetitions of the same frequency on two adjacent tone-pips.

The bulk of the analysis (below) is focused on understanding whether, in addition to these global effects of regularity on the responses to the sequence, responses to the outlier tones are also affected.

#### 3.1.3 Deviance-evoked EEG responses

For quantifying the deviance response (response to the outlier relative to the no-outlier conditions), data were high-pass filtered at 2 Hz so as to remove the sustained response difference between REG and RAND sequences and focus on brain activity specifically evoked by the frequency outliers. A comparison between REGno and RANDno confirmed no difference between these conditions after filtering.

The outlier-evoked responses (Fig. 2a) were comprised of a series of peaks closely resembling the standard N1—P2—N2 sequence commonly observed at stimulus onset, or for changes within ongoing sounds (Martin & Boothroyd, 2000). To quantify the effect of context on the response to the outlier, we first identified the channels and time intervals that show a response to the outlier (**main effect of deviance ROI**), we then investigated how this ROI is affected by context regularity (**effect of regularity**) by comparing outlier responses in REG versus RAND contexts.

To identify the **main effect of deviance ROI**; channels and time-intervals showing a response to the outlier, collapsed across REG or RAND context, were identified (see ‘Methods’). This allowed separation of neural activity associated with the ongoing context of the sequence from those strictly evoked by the outlier tone. The resulting three ROIs, shown in Fig. 2b, correspond to the peaks observed in the time domain (Fig. 2a). **ROI1** comprised thirty-nine fronto-central channels which show a significant negativity between 80 and 145 msec (*p* = .001), corresponding most closely in time and topography to the N1. **ROI2**, a cluster of 33 channels at 165–245 msec (*p* = .001), had a similar topography but with a positive polarity, **ROI3**, from 290 to 320 msec (*p* = .016), had a smaller spatial extent (10 channels) and negative polarity (Fig. 2c).

To quantify the effect of regularity on the outlier response, a comparison between deviance responses in REG relative to RAND was then calculated for each of the 3 ROIs identified above (see methods). In ROI1, a subset (21 channels) showed an effect of regularity on the outlier response (*p* = .005), which was 71% larger (calculated over mean activity within the significant channels), in REG sequences. In ROI2, responses were also larger (by 41%) in REG (*p* = .002) in a subset of 17 channels (Fig. 2d). There was no effect of regularity in ROI3. Importantly, since the analysis above is performed on high pass filtered, and baselined, data, the effect of regularity on the deviance response occurs over and above the sustained response difference between the two sequence types (see below).

Contrasts were also computed in source space (see methods), both for the **main effect of deviance**, and for the **effect of regularity**. The **main effect of deviance** in ROI1 was localised to bilateral temporal cortex (Fig. 2e, top), maximal in right superior/middle frontal gyrus (S/MFG) with a peak T-statistic of 3.05. In ROI2, the main effect of deviance was associated with temporal lobe activation, but this time more prominently left-lateralised as well as situated more frontally around the temporal pole (TP), with a peak of T = 3.55 in the left middle temporal gyrus. Right-hemisphere activation is seen around the intraparietal sulcus (IPS) and the central sulcus (CS; Fig. 2e, bottom).

For the **effect of regularity** (Fig. 2f, top), in ROI1 we observed increased deviance response in REG at right TP and right orbital gyrus (OG), where the maximal t-statistic of 2.86 was observed. In ROI2, REGo elicited a greater deviance response than RANDo in left temporal cortex, with a peak T-statistic of 3.05 in left middle temporal gyrus (MTG) and superior temporal sulcus (STS). Increased activity was also seen in right S/MFG.

#### 3.1.4 Offset-evoked EEG responses

Interestingly, an effect of regularity is also present during the offset response, which is seen from about 50 msec after the cessation of the sequence (Fig. 3a). The offset peak was compared between REG and RAND (pooling across trials which contained and did not contain a violation; high pass filtered to remove differences associated with the sustained response) using the same clustering approach as above. REG showed a significantly larger offset response than RAND, from 85 to 175 msec (*p* < .001) in most channels (more negative in a fronto-central cluster of 58 channels, *p* < .001; and more positive in a temporal-occipital cluster of 50 channels, *p* < .001). There was also a significantly more positive response from 215 to 300 msec (*p* = .008) post-offset in a fronto-central cluster of 41 channels (Fig. 3b; lower right). Statistical comparison was performed at each time-point and channel, but for illustrative purposes the time-domain response averaged over the 58 channels in the first negative cluster, is shown in Fig. 3b.

Overall, the EEG results demonstrate that the brain rapidly detects the structure within REG and RAND sequences and is sensitive to the uncertainty induced by the sensory context, such that (frequency or offset) violations within a volatile (less predictable) RAND context are considered less surprising than identical events within a stable, predictable, background.

### 3.2 Experiment 2 – behavioural sensitivity to frequency-outliers in REG and RAND sequences

We measured listeners' ability to detect frequency outliers in matched REG and RAND sequences (Fig. 1a,b). The mean reaction time to outlier tones in the control condition was 329 ± 16 msec, giving an estimate of participants' basic response time. The mean reaction times to outliers within REG and RAND were 347 ± 15 msec and 387 ± 25 msec, respectively. Paired-sample *t*-tests were carried out on the subject-wise averages of both RT and d’ for REG versus RAND. Reaction times were significantly faster (*p* = .01) and sensitivity (d’) significantly higher (*p* < .001) to outliers in REG, versus RAND sequences. See Fig. 1c.

To summarise, despite carefully matched properties of the regular and random stimuli used, we observe robustly greater behavioural sensitivity, as well as faster reaction times, to outlier tones which violate a regular sequence.

## 4 Discussion

We investigated whether and how the predictability of successive events within rapid tone-pip sequences influences responses to deviant tones. Whilst it is commonly observed that regularity shapes responses to standards, even in complex sequences (Heilbron and Chait, 2017, Khouri and Nelken, 2015), effects on the response to the deviant itself have been more elusive. For example, Yaron, Hershenhoren, and Nelken (2012) report remarkable sensitivity to the temporal patterning of long sound sequences, but these effects are revealed via changes to the response to the standard, but not deviant sounds. Similarly, Costa-Faidella, Baldeweg, Grimm, and Escera (2011) showed robust effects of regularity on the standard, such that more repetition suppression is seen in a temporally regular than a jittered context - but the response to the deviant itself did not differ (see also Christianson, Chait, de Cheveigné, & Linden, 2014).

Here, replicating our previous work (Barascud et al., 2016, Southwell et al., 2017) we observed substantial effects of context (REG *vs* RAND) on the brain response to the sequence. Following the discovery of the regularity, REG elicited a higher sustained response. Importantly, we further demonstrate sizeable effects of sequence context specifically on the response to the deviant. Our results reveal two main findings: *Firstly*, robust effects of context were observed despite the fact that patterns were never repeated and had to be discovered anew on each trial. Though the outlier is set apart in frequency from the range defined by the sequence, and can in principle be detected based on this information alone, its detection was facilitated by sequence context. This was revealed in behaviour (Experiment 2) and in EEG responses from naïve distracted listeners (Experiment 1) where frequency outliers within regular sequences evoked a larger response (from 80 msec after outlier onset) than matched outliers in random sequences. *Secondly*, the neural sources which underlie the effect of regularity, are, at least in part, distinct from those activated by the main effect of deviance (collapsed across REG *vs* RAND context). Whilst the latter was associated with the standard temporo-frontal network commonly implicated in frequency-based deviance detection, the effect of regularity was underpinned by sources in right temporal pole and orbitofrontal cortex.

The implications of these findings to our understanding of how the brain tracks and represents unfolding structure in rapid sensory signals are discussed, in turn below.

### 4.1 Automatic tracking of sensory sequence structure

Previous reports in the MMN (Paavilainen, 2013, Bendixen et al., 2012), statistical learning (Koelsch et al., 2000) and music processing literature (Maess et al., 2001) have demonstrated increased responses to deviants within structured contexts, relative to random contexts. For example, in a study of musical expectation, Pearce et al. (2010) showed that low probability notes, compared to high probability notes, elicited a larger negative component at around 400 msec. Using non-musical, abstract tone sequences arranged in a random or ascending frequency pattern, Vaz Pato et al. (2002) demonstrated increased MMN responses to frequency deviants within the structured sequences. Koelsch et al. (2016) further showed increased negativity (from 130 to 220 msec post onset) to less probable items within sequences of tones with specifically controlled transition probabilities. Furl et al. (2011) trained participants to discriminate Markov sequences of pure tones from random ones and demonstrated a difference between low and high probability tones from 200 msec post-onset (during the P2 peak) originating in the right temporo-parietal junction.

However, a limiting factor in generalizing those results to listening in natural environments is the use of regularities established over an extended period. For example, a fixed pattern or transition probability matrix throughout the experiment; or even, for music, over a lifetime. As a consequence, these paradigms might be tapping long-term memory mechanisms; fundamentally different from those implicated in processing rapidly evolving and novel sensory sequences. Furthermore, brain activity was often recorded while participants were required to make decisions about the predictability of the pattern (Furl et al., 2011, Pearce et al., 2010) possibly implicating mechanisms related to active, overt tracking of sequence structure.

To probe rapid, automatic and pre-attentive processes associated with tracking evolving sensory statistics in the environment, we used rapid tone patterns (20 Hz); beyond the rate which human listeners can actively track (Warren et al., 1991, Warren and Obusek, 1972). Unique sequences, whether REG or RAND, were used on each trial and (in Experiment 1) participants were kept naïve about the stimuli. We show that even when the regularity must be detected and represented afresh each trial, the response to a deviant is immediately modulated.

The deviant responses seen here - a standard succession of N1—P2—N2 deflections - are similar to those commonly observed in the human Stimulus-Specific Adaptation (SSA) literature (Briley and Krumbholz, 2013, Herrmann et al., 2013) and which have previously been shown to be affected by both simple adaptation (repetition suppression) as well as more complex statistical context (relative probability of the deviant; Herrmann et al., 2013). Here we demonstrate a substantially larger response (71% increase in the first window) in REG relative to RAND sequences, confirming that these early deviant-evoked responses are also subject to automatic modulation by the degree of predictability in the ongoing sequence context.

In a separate experiment (Experiment 2), the effect of regularity was also revealed behaviourally - listeners are faster and substantially more accurate at detecting outlier tones within regularly repeating (REG), relative to random (RAND) tone-pip sequences, despite matched frequency content.

These findings are consistent with the notion that the brain continually tracks and maintains a detailed representation of the structure of the unfolding sensory input and that this representation shapes the processing of incoming information: deviants within high-precision sequences evoke higher prediction errors than identical events embedded in matched sequences of lower precision. A conceptually similar explanation may be framed in the context of perceptual binding: the tones in REG sequences are bound together by virtue of the underlying regularity model (Winkler et al., 2009, Andreou et al., 2011), such that deviants, not confirming to the rule, are perceptually represented as distinct ‘objects’ and therefore evoke a larger neural response.

An alternative explanation for the observed findings might have been that regular patterns automatically attract attention (Zhao, Al-Aidroos, & Turk-Browne, 2013), and that this facilitates the detection of deviants in REG sequences. Southwell et al. (2017) directly investigated the question of whether attention is biased towards REG sequences (essentially identical to those used here), and found no attentional bias towards either REG or RAND. The fact that when interrogated, participants in the present study did not report noticing a distinction between REG and RAND trials also supports the conclusion that attention is not a likely explanation for the observed pattern of effects. Furthermore, the effects of attention on deviance detection are commonly associated with the presence of a P300 response (Chennu and Bekinschtein, 2012, Molloy et al., 2015) reflecting the fact that the deviant was consciously perceived. The P300 was absent here. Instead our results point to an early and time-limited (between 80 and 250 msec) effect of context on the deviant response.

We also observed a remarkably strong effect of regularity on the offset response to the sequences. An offset is a special case of deviance, reflecting the violation of the expectation that a tone will be presented. This effect has been studied extensively in the context of the auditory omission (Chennu et al., 2016, Phillips et al., 2016) or offset (Andreou, Griffiths, & Chait, 2015) paradigms, where an evoked response occurs to unexpected omissions of sounds, at a similar latency to the early responses to actual sounds, but only when the preceding sequence allowed a prediction to be formed about the omitted tone's properties. That both frequency and offset deviants are affected by regularity is consistent with the notion that the overall predictability of the pattern (the precision of the prediction the observer can make about an upcoming event) affects error responses regardless of the dimension in which the deviance occurs.

### 4.2 Source reconstruction

The main effect of deviance, computed by collapsing over sequence context and hence assumed to reflect the mismatch in frequency, was significant across a central subset of channels commonly associated with auditory responses (Fig. 2c). In line with the standard network of bilateral auditory and right-hemisphere frontal sources often implicated in pre-attentive deviance detection (Doeller et al., 2003, Garrido et al., 2009, Halgren et al., 2010, Opitz et al., 2002), source analysis suggested that activity within ROI1 (80–145 msec) originated in temporal cortex and right prefrontal cortex. Later, in ROI2 (165–245 msec), the anterior portion of the left temporal cortex showed the strongest deviant-evoked response, with some additional activation in right intraparietal sulcus (IPS). The IPS is commonly implicated in auditory perceptual organisation (Cusack, 2005) and specifically figure-ground segregation (Teki et al., 2016) and its involvement here may be linked to processes which stream the deviant tone away from the ongoing sequence.

The increased deviance response in REG sequences (‘effect of regularity’) was associated with regions that are, at least in part, distinct from those involved in coding for the main effect of deviance. This was observed both in source space and in channel space, where the effect of regularity was only significantly present in a frontal subset of the channels identified as sensitive to the outlier.

In source space, the effect of regularity in ROI1 is underpinned by activity in the right temporal pole and right orbitofrontal cortex. This is in contrast to the main effect of deviance which is dominated by extensive activation of temporal areas. The right temporal pole and right orbitofrontal cortex have previously been implicated in sensitivity to context: the right anterior temporal cortex has been shown to be sensitive to the level of disorder in auditory and visual sequences, demonstrating higher activity the more ordered the sequence (Nastase, Iacovella, & Hasson, 2014). Orbitofrontal cortex has been proposed to be a source of top-down modulation on auditory cortex according to context (Frey, Kostopoulos, & Petrides, 2004) and is more generally implicated in integrating top-down priors with current information (Kepecs et al., 2008, Nogueira et al., 2017, Payzan-LeNestour et al., 2013, Wilson et al., 2014). The present results provide converging evidence for the role of these areas, outside of the standard deviance-detection network, in monitoring sequence structure.

Overall, source results replicate the ubiquitous network of bilateral auditory cortex and right pre-frontal sources as underpinning frequency-based deviance detection and additionally implicate the temporal pole as well as right orbitofrontal and pre-frontal cortex in nuancing these responses according to the preceding sequence context. This suggests that simple deviance responses are underpinned by activity in auditory cortex whereas more complex sequence structure related information is maintained outside of auditory cortex within frontal areas.

Source reconstruction based on EEG, particularly in the absence of individualised head-models, must be interpreted with caution. Future work, using more sensitive source-imaging, is required to understand and elaborate on these processes.

### 4.3 Implications for theories of predictive coding

All the deviant effects observed here were superimposed on an overall higher sustained response to REG relative to RAND patterns. A specific mechanistic account for the increased sustained response remains elusive, but previous work has demonstrated that the amplitude of the sustained response is related to the predictability or precision of the ongoing acoustic pattern (Auksztulewicz et al., 2017, Barascud et al., 2016, Sohoglu and Chait, 2016, Southwell et al., 2017), such that increased predictability is systematically associated with higher sustained responses. This effect, underpinned by increased activity in a network of temporal, frontal and hippocampal sources (Auksztulewicz et al., 2017, Barascud et al., 2016), may reflect a mechanism which tracks the context-dependent reliability of sensory streams.

Over and above this context effect, we demonstrated modulation of deviant specific responses. Though the present experiments do not provide evidence for a concrete link between the sustained response and the deviant response, they may be interpreted as reflecting two aspects of predictive coding. According to predictive coding theory, surprise is determined by two processes: *prediction error* evoked by a stimulus that differs from expectations, and also the *precision* associated with the input; i.e., the reliability attributed to the sensory stream (Heilbron and Chait, 2017, Kanai et al., 2015). It is hypothesized that brain responses to predictable (highly precise) stimuli are up-weighted (e.g., through gain modulation) to focus perception on stable features of the environment (Feldman & Friston, 2010). It is tempting to interpret the increased amplitude of the sustained response to regular sequences as a manifestation of precision-weighting (Auksztulewicz et al., 2017, Barascud et al., 2016, Sohoglu and Chait, 2016, Southwell et al., 2017), though it remains unclear whether the sustained effects seen here are indeed excitatory (as the gain modulation postulated by predictive coding; see further discussion in Southwell et al., 2017).

Importantly, the pattern of results we observe is not fully consistent with the standard predictive coding account of ‘prediction error’. Source analysis suggests the response to deviants in regular sequences was not merely enhanced relative to matched deviants in random sequences but rather arose in part via the involvement of distinct underlying sources. Therefore, an account in terms of differential precision weighing over the same prediction error units, as proposed by predictive coding (Feldman and Friston, 2010, Kanai et al., 2015), may not fully account for the observed effects. Instead, the results point to a model where increasingly complex aspects of the same violating event are encoded in progressively higher stages of the processing hierarchy. In the deviance responses studied here this was revealed by predominantly auditory cortical sources coding for frequency deviance and frontal sources encoding more complex properties of pattern violation.

## References

[bib1] Aghamolaei M., Zarnowiec K., Grimm S., Escera C. (2016). Functional dissociation between regularity encoding and deviance detection along the auditory hierarchy. The European Journal of Neuroscience.

[bib2] Andreou L.-V., Griffiths T.D., Chait M. (2015). Sensitivity to the temporal structure of rapid sound sequences — an MEG study. Neuroimage.

[bib0089] Andreou L.-V., Kashino M., Chait M. (2011). The role of temporal regularity in auditory segregation. Hearing Research.

[bib3] Auksztulewicz R., Barascud N., Cooray G., Nobre A.C., Chait M., Friston K. (2017). The cumulative effects of predictability on synaptic gain in the auditory processing stream. Journal of Neuroscience.

[bib4] Ayala Y.A., Pérez-González D., Malmierca M.S. (2016). Stimulus-specific adaptation in the inferior colliculus: The role of excitatory, inhibitory and modulatory inputs. Biological Psychology.

[bib5] Barascud N., Pearce M.T., Griffiths T.D., Friston K.J., Chait M. (2016). Brain responses in humans reveal ideal observer-like sensitivity to complex acoustic patterns. Proceedings of the National Academy of Sciences of the United States of America.

[bib0084] Bendixen A., Schröger E., Ritter W., Winkler I. (2012). Regularity extraction from non-adjacent sounds. Frontiers in Physiology.

[bib6] Bestmann S., Harrison L.M., Blankenburg F., Mars R.B., Haggard P., Friston K.J. (2008). Influence of uncertainty and surprise on human corticospinal excitability during preparation for action. Current Biology.

[bib7] Briley P.M., Krumbholz K. (2013). The specificity of stimulus-specific adaptation in human auditory cortex increases with repeated exposure to the adapting stimulus. Journal of Neurophysiology.

[bib9] Chennu S., Bekinschtein T.A. (2012). Arousal modulates auditory attention and awareness: Insights from sleep, sedation, and disorders of consciousness. Frontier of Psychology.

[bib10] Chennu S., Noreika V., Gueorguiev D., Shtyrov Y., Bekinschtein T.A., Henson R. (2016). Silent expectations: Dynamic causal modeling of cortical prediction and attention to sounds that weren't. Journal of Neuroscience.

[bib11] Christianson G.B., Chait M., de Cheveigné A., Linden J.F. (2014). Auditory evoked fields measured noninvasively with small-animal MEG reveal rapid repetition suppression in the Guinea pig. Journal of Neurophysiology.

[bib12] Costa-Faidella J., Baldeweg T., Grimm S., Escera C. (2011). Interactions between “what” and “when” in the auditory system: Temporal predictability enhances repetition suppression. The Journal of Neuroscience.

[bib13] Cusack R. (2005). The intraparietal sulcus and perceptual organization. Journal of Cognitive Neuroscience.

[bib14] Daikhin L., Ahissar M. (2012). Responses to deviants are modulated by subthreshold variability of the standard. Psychophysiology.

[bib15] Dale A.M., Liu A.K., Fischl B.R., Buckner R.L., Belliveau J.W., Lewine J.D. (2000). Dynamic statistical parametric mapping: Combining fMRI and MEG for high-resolution imaging of cortical activity. Neuron.

[bib16] Dayan P., Hinton G.E., Neal R.M., Zemel R.S. (1995). The Helmholtz machine. http://dxdoiorg/101162/neco199575889.%207:889%5f904.

[bib17] Doeller C.F., Opitz B., Mecklinger A., Krick C., Reith W., Schröger E. (2003). Prefrontal cortex involvement in preattentive auditory deviance detection. Neuroimage.

[bib18] Efron B., Tibshirani R.J. (1993).

[bib19] Feldman H., Friston K.J. (2010). Attention, uncertainty, and free-energy. Frontiers in Human Neuroscience.

[bib20] Frey S., Kostopoulos P., Petrides M. (2004). Orbitofrontal contribution to auditory encoding. Neuroimage.

[bib21] Friston K. (2005). A theory of cortical responses. Philosophical Transactions of the Royal Society B Biological Sciences.

[bib22] Friston K., Kiebel S. (2009). Predictive coding under the free-energy principle. Philosophical Transactions of the Royal Society London B Biological Science.

[bib23] Fuchs M., Kastner J., Wagner M., Hawes S., Ebersole J.S. (2002). A standardized boundary element method volume conductor model. Clinical Neurophysiology.

[bib24] Furl N., Kumar S., Alter K., Durrant S., Shawe-Taylor J., Griffiths T.D. (2011). Neural prediction of higher-order auditory sequence statistics.

[bib25] Garrido M.I., Friston K.J., Kiebel S.J., Stephan K.E., Baldeweg T., Kilner J.M. (2008). The functional anatomy of the MMN: A DCM study of the roving paradigm. Neuroimage.

[bib26] Garrido M.I., Kilner J.M., Stephan K.E., Friston K.J. (2009). The mismatch negativity: A review of underlying mechanisms. Clinical Neurophysiology.

[bib27] Garrido M.I., Sahani M., Dolan R.J. (2013). Outlier responses reflect sensitivity to statistical structure in the human brain. PLoS Computational Biology.

[bib28] Grill-Spector K., Henson R., Martin A. (2006). Repetition and the brain: Neural models of stimulus-specific effects. Trends in Cognitive Sciences.

[bib29] Gross J., Baillet S., Barnes G.R., Henson R.N., Hillebrand A., Jensen O. (2013). Good practice for conducting and reporting MEG research. Neuroimage.

[bib31] Halgren E., Sherfey J., Irimia A., Dale A.M., Marinkovic K. (2010). Sequential temporo-fronto-temporal activation during monitoring of the auditory environment for temporal patterns. Human Brain Mapping.

[bib32] Heilbron M., Chait M. (2017). Great expectations: Is there evidence for predictive coding in auditory cortex?. Neuroscience.

[bib33] Herrmann B., Henry M.J., Fromboluti E.K., McAuley J.D., Obleser J. (2015). Statistical context shapes stimulus-specific adaptation in human auditory cortex. Journal of Neurophysiology.

[bib34] Herrmann B., Henry M.J., Obleser J. (2013). Frequency-specific adaptation in human auditory cortex depends on the spectral variance in the acoustic stimulation. Journal of Neurophysiology.

[bib36] Kanai R., Komura Y., Shipp S., Friston K. (2015). Cerebral hierarchies: Predictive processing, precision and the pulvinar. Philosophical Transactions of the Royal Society B Biological Sciences.

[bib37] Kepecs A., Uchida N., Zariwala H.A., Mainen Z.F. (2008). Neural correlates, computation and behavioural impact of decision confidence. Nature.

[bib38] Khouri L., Nelken I. (2015). Detecting the unexpected. Current Opinion in Neurobiology.

[bib39] Kleiner M., Brainard D., Pelli D., Ingling A. (2007). What's new in Psychtoolbox-3. Presented at the perception 36 ECVP abstract supplement.

[bib40] Koelsch S., Busch T., Jentschke S., Rohrmeier M. (2016). Under the hood of statistical learning: A statistical MMN reflects the magnitude of transitional probabilities in auditory sequences. Scientific Reports.

[bib41] Koelsch S., Gunter T., Friederici A.D., Schröger E. (2000). Brain indices of music processing: “nonmusicians” are musical. Journal of Cognitive Neuroscience.

[bib42] Maess B., Koelsch S., Gunter T.C., Friederici A.D. (2001). Musical syntax is processed in broca's area: An MEG study. Nature Neuroscience.

[bib43] Maris E., Oostenveld R. (2007). Nonparametric statistical testing of EEG- and MEG-data. Journal of Neuroscience Methods.

[bib44] Martin B.A., Boothroyd A. (2000). Cortical, auditory, evoked potentials in response to changes of spectrum and amplitude. The Journal of the Acoustical Society of America.

[bib45] May P.J.C., Tiitinen H. (2010). Mismatch negativity (MMN), the deviance-elicited auditory deflection, explained. Psychophysiology.

[bib46] McDermott J.H., Schemitsch M., Simoncelli E.P. (2013). Summary statistics in auditory perception. Nature Neuroscience.

[bib47] Molloy K., Griffiths T.D., Chait M., Lavie N. (2015). Inattentional deafness: Visual load leads to time-specific suppression of auditory evoked responses. The Journal of Neuroscience.

[bib48] Näätänen R., Alho K. (1995). Mismatch negativity--a unique measure of sensory processing in audition. International Journal of Neuroscience.

[bib49] Nastase S., Iacovella V., Hasson U. (2014). Uncertainty in visual and auditory series is coded by modality-general and modality-specific neural systems. Human Brain Mapping.

[bib51] Nelken I. (2014). Stimulus-specific adaptation and deviance detection in the auditory system: Experiments and models. Biological Cybernetics.

[bib52] Nogueira R., Abolafia J.M., Drugowitsch J., Balaguer-Ballester E., Sanchez-Vives M.V., Moreno-Bote R. (2017). Lateral orbitofrontal cortex anticipates choices and integrates prior with current information. Nature Communications.

[bib53] Oostenveld R., Fries P., Maris E., Schoffelen J.-M. (2010). FieldTrip: Open source software for advanced analysis of MEG, EEG, and invasive electrophysiological data. Computational Intelligence and Neuroscience.

[bib54] Opitz B., Rinne T., Mecklinger A., Cramon von D.Y., Schröger E. (2002). Differential contribution of frontal and temporal cortices to auditory change detection: fMRI and ERP results. Neuroimage.

[bib55] Paavilainen P. (2013). The mismatch-negativity (MMN) component of the auditory event-related potential to violations of abstract regularities: A review. International Journal of Psychophysiology.

[bib56] Parras G.G., Nieto-Diego J., Carbajal G.V., Valdés-Baizabal C., Escera C., Malmierca M.S. (2017). Neurons along the auditory pathway exhibit a hierarchical organization of prediction error. Nature Communications.

[bib57] Payzan-LeNestour E., Dunne S., Bossaerts P., O'Doherty J.P. (2013). The neural representation of unexpected uncertainty during value-based decision making. Neuron.

[bib58] Pearce M.T., Ruiz M.H., Kapasi S., Wiggins G.A., Bhattacharya J. (2010). Unsupervised statistical learning underpins computational, behavioural, and neural manifestations of musical expectation. Neuroimage.

[bib59] Phillips H.N., Blenkmann A., Hughes L.E., Kochen S., Bekinschtein T.A., CAN C. (2016). Convergent evidence for hierarchical prediction networks from human electrocorticography and magnetoencephalography. Cortex.

[bib60] Rubin J., Ulanovsky N., Nelken I., Tishby N. (2016). The representation of prediction error in auditory cortex. PLoS Computational Biology.

[bib61] Saffran J.R., Aslin R.N., Newport E.L. (1996). Statistical learning by 8-month-old infants. Science.

[bib62] Saffran J.R., Johnson E.K., Aslin R.N., Newport E.L. (1999). Statistical learning of tone sequences by human infants and adults. Cognition.

[bib64] Sohoglu E., Chait M. (2016). Detecting and representing predictable structure during auditory scene analysis. Elife.

[bib65] Southwell R., Baumann A., Gal C., Barascud N., Friston K., Chait M. (2017). Is predictability salient? A study of attentional capture by auditory patterns. Philosophical Transactions of the Royal Society London B Biological Sciences.

[bib66] Taaseh N., Yaron A., Nelken I. (2011). Stimulus-specific adaptation and deviance detection in the rat auditory cortex. PLoS One.

[bib67] Tanner W.P., Swets J.A. (1954). A decision-making theory of visual detection. Psychological Review.

[bib68] Teki S., Barascud N., Picard S., Payne C., Griffiths T.D., Chait M. (2016). Neural correlates of auditory figure-ground segregation based on temporal coherence. Cerebral Cortex.

[bib70] Turk-Browne N.B., Scholl B.J., Chun M.M., Johnson M.K. (2009). Neural evidence of statistical learning: Efficient detection of visual regularities without awareness. Journal of Cognitive Neuroscience.

[bib71] Ulanovsky N., Las L., Nelken I. (2003). Processing of low-probability sounds by cortical neurons. Nature Neuroscience.

[bib72] Vaz Pato M., Jones S.J., Perez N., Sprague L. (2002). Mismatch negativity to single and multiple pitch-deviant tones in regular and pseudo-random complex tone sequences. Clinical Neurophysiology.

[bib73] Warren R.M., Gardner D.A., Brubaker B.S., Bashford J.A. (1991). Melodic and nonmelodic sequences of tones: Effects of duration on perception. Music Perception Interdisciplinary Journal.

[bib74] Warren R.M., Obusek C.J. (1972). Identification of temporal order within auditory sequences. Perception and Psychophysics.

[bib0094] Wilson R.C., Takahashi Y.K., Schoenbaum G., Niv Y. (2014). Orbitofrontal cortex as a cognitive map of task space. Neuron.

[bib76] Winkler I., Denham S.L., Nelken I. (2009). Modeling the auditory scene: Predictive regularity representations and perceptual objects. Trends in Cognitive Sciences.

[bib78] Yaron A., Hershenhoren I., Nelken I. (2012). Sensitivity to complex statistical regularities in rat auditory cortex. Neuron.

[bib79] Zhao J., Al-Aidroos N., Turk-Browne N.B. (2013). Attention is spontaneously biased toward regularities. Psychological Science.

